# Orthodontic Management in Patients with Amelogenesis Imperfecta: A Narrative Review

**DOI:** 10.3390/reports9040321

**Published:** 2026-09-23

**Authors:** Amalia Syrioti, Faidra Chatzikallinikidou, Christina Charisi, Filippos Fytros, Nikolaos Spantidakis, Panagiota Dimitropoulou, Konstantinos Poulopoulos, Stefanos Zisis, Athanasios Poulopoulos, Vasileios Zisis

**Affiliations:** 1 Department of Dentistry, School of Dentistry, European University Cyprus, Nicosia 2404, Cyprus; 2 Department of Oral Medicine and Pathology, School of Dentistry, Aristotle University of Thessaloniki, 54124 Thessaloniki, Greeceakpoul@dent.auth.gr (A.P.); 3 Department of Dentoalveolar Surgery, Surgical Implantology and Radiology, School of Dentistry, Aristotle University of Thessaloniki, 54124 Thessaloniki, Greece; 4 Clinic for Operative Dentistry, Periodontology and Preventive Dentistry, University Hospital RWTH Aachen, 52074 Aachen, Germany

**Keywords:** amelogenesis imperfecta, orthodontic treatment, orthodontic appliances, orthodontics, braces, aligners

## Abstract

**Background**: Amelogenesis imperfecta affecting enamel formation is frequently associated with a variety of malocclusions and eruption disturbances. Orthodontic treatment is crucial in comprehensive patient management. Previous studies have shown that orthodontic treatment becomes more challenging in patients with AI as the defective enamel affects the bracket bonding, retention of appliances and treatment mechanics and often needs modified bonding protocols, pre-treatment restorative build-ups, skeletal anchorage and general multidisciplinary management. There is no evidence of the ideal orthodontic treatment yet; available evidence only consists of case reports and small observational studies that address specific aspects with no established consensus. Therefore, this narrative review aimed to critically examine the contemporaneous evidence on orthodontic assessment and treatment planning in AI patients, drawing on studies identified through a structured literature search. **Materials and Methods**: An electronic search of the literature was conducted in the PubMed, Scopus, and ScienceDirect databases. Out of 640 records that were initially identified, 11 studies met the inclusion criteria for qualitative synthesis. **Results**: Bonding issues resulting from enamel defects tend to be identified as a significant clinical challenge necessitating modified orthodontic techniques. The included studies demonstrated that by using individualized treatment planning, modified bonding procedures, fixed appliances, expansion procedures, skeletal anchorage and combined orthognathic surgery in some cases within the multidisciplinary treatment approach, there can be successful orthodontic management for these patients. **Conclusions**: Vital steps such as early diagnosis, careful treatment planning, and coordination with restorative and prosthodontic treatment were associated with better functional and esthetic outcomes. Consequently, even though there is limited evidence, the available literature supports a customized interdisciplinary approach as the most predictable and effective strategy for managing orthodontic problems in patients with AI, while emphasizing that case reports and small observational studies are the main sources of current findings.

## 1. Introduction

Amelogenesis imperfecta (AmI) is a rare genetic disorder that disrupts the formation of tooth enamel, affecting both primary and permanent teeth. The condition is clinically variable and can be clinically divided based on how it affects the enamel. Teeth affected by AmI can present with thin, pitted, rough, discolored, hypoplastic, hypomineralized or even rapidly wearing enamel [1]. Patients commonly experience tooth sensitivity, poor esthetics, enamel breakdown, eating difficulties and the need for long-term dental care [2]. These clinical issues caused by AmI can affect the function of the oral cavity, its appearance, quality of life, and psychosocial wellbeing, especially in children and adolescents [2].

Orthodontics plays a significant role in the management of Aml as many patients affected by the condition present with malocclusion, open bite, spacing, crowding, transverse deficiency, cross-bite, or eruption disturbances [3]. Analyses have reported that open bite and eruption disturbances were among the most common orthodontic findings in patients with Aml, each affecting 36.8% of their sample [3]. Therefore, orthodontic treatment can be crucial for the management of these cases. For some cases, orthodontic treatment is used directly to improve tooth alignment and occlusion, while in others it is part of a wider interdisciplinary plan before restorative, prosthodontic or surgical rehabilitation [4,5,6].

However, orthodontic treatment in patients with Aml can be challenging for multiple reasons. Defective enamel may reduce bracket bond strength and increase the risk of repeated bracket failure [4,5]. Fixed appliances may require modified bonding approaches, rebonding procedures, bonding to provisional restorations, or even the use of crowns before orthodontic treatment to improve appliance retention [4,7]. In more complex cases, orthodontic management may also involve expansion appliances, skeletal anchorage, multiloop edgewise archwire mechanics, orthognathic surgery or compression osteogenesis [6,8,9].

This shows that orthodontic treatment in patients with Aml patients must be carefully planned according to enamel type, age, severity of malocclusion, restorative needs and long-term stability [4,10].

Differentiating Aml from other developmental dental disorders that might have similar enamel defects is an additional clinical element. This is especially significant in pediatric dentistry because molar-incisor hypomineralization (MIH) might be misinterpreted for milder or limited enamel abnormalities [11,12]. Although enamel discoloration, sensitivity, hypomineralization and structural breakdown might be associated with both Aml and MIH, their typical distribution and underlying etiology are dissimilar [12,13]. While MIH usually affects one or more first permanent molars and may be linked to involvement of the permanent incisors, Aml typically affects enamel throughout the dentition and may involve both primary and permanent teeth [11,12,13]. Precisely differentiating between these diseases is essential because it may impact the design of orthodontic, restorative and preventive treatments [11,13,14,15]. From the orthodontic perspective, the widespread enamel involvement and structural fragility associated with AmI may present specific challenges for appliance bonding and retention that are generally less pronounced in patients with MIH [4,7].

Other hereditary dental disorders to be considered in the differential diagnosis include dentinogenesis imperfecta and dentin dysplasia, which mainly involve the dentin and not the enamel [11,12,13,14]. Clinical examination, radiographic assessment, family history and where appropriate genetic evaluation may thus contribute to establishing an accurate diagnosis and identifying associated dental and craniofacial characteristics.

Despite the clinical importance of this topic, the available evidence on orthodontic management of Aml remains limited [3,16]. Most of the existing literature consists of case reports, clinical reports and small observational studies rather than controlled clinical trials which make it difficult to establish a single, standardized and optimal orthodontic protocol for patients with Aml [14,15]. Therefore, important aspects of orthodontic management such as bonding strategies, appliance selection, treatment timing, and management of transverse and vertical discrepancies as well as coordination with restorative or surgical treatment are still insufficiently clarified in the literature. This knowledge gap prompted the present narrative review [16].

The aim of this narrative review was to critically appraise the existing evidence regarding the orthodontic management of patients with AmI, with particular focus on the clinical challenges related to enamel defects, orthodontic bonding and appliance retention, malocclusion and eruption disturbances, expansion and interceptive treatment, and multidisciplinary orthodontic-restorative and orthodontic-surgical approaches. The review also sought to identify gaps in the current evidence base and to provide a clinically oriented synthesis of the available treatment strategies.

## 2. Materials and Methods

### 2.1. Eligibility Criteria

The Population, Intervention, Comparison, Outcomes and Study design (PICOS) framework was applied to formulate the eligibility requirements (Table 1).

Studies were considered eligible if they reported:

- Patients with a confirmed diagnosis of amelogenesis imperfecta (Aml).
- Patients for whom an orthodontic evaluation or treatment plan had been established and who subsequently underwent orthodontic treatment or orthodontic management.
- Clinical studies such as case reports, case series and observational studies.
- Studies published between 2016 and 2026.

Studies were excluded if they:

- Did not include patients with a confirmed diagnosis of amelogenesis imperfecta (Aml).
- Did not report orthodontic evaluation, treatment or management.
- Were in vitro or animal studies.
- Were review articles, systematic reviews or a meta-analysis.
- Did not report sufficient information regarding orthodontic treatment or management.
- Lacked adequate clinical information relevant to the objectives of the review.

### 2.2. PICOS Framework

Table 1 describes the PICOS Framework which is used to determine the eligibility criteria of the present study.

**Table 1:** PICOS framework used to determine the eligibility criteria of the present study.

Component	Description
Population (P)	Individuals with confirmed diagnosis of Amelogenesis imperfecta (Aml).
Intervention (I)	Orthodontic evaluation, treatment or management, including conventional and interdisciplinary orthodontic approaches
Comparison (C)	Different orthodontic techniques or treatment approaches, where reported; no comparison was required for case reports and case series
Outcomes (O)	Orthodontic assessment, treatment planning, clinical management, orthodontic procedures, treatment outcomes, and reported clinical complications
Study Design (S)	Clinical studies, including case reports, case series and observational studies.

### 2.3. Information Sources and Search Strategy

Three databases (PubMed, Scopus, and ScienceDirect) were searched electronically. Publications from 2016 to 2026 were the primary focus of the search. Keywords related to amelogenesis imperfecta and orthodontic management were combined to construct the search strategy. The primary keyword phrase was “amelogenesis imperfecta” in conjunction with terms pertaining to orthodontics including “orthodontic”, “braces”, “aligners”, “orthodontic treatment”, and “orthodontic appliances”.

Search methods specific to each database were adjusted according to its requirements. PubMed employed keywords contained within the title/abstract field, whereas Scopus utilized the TITLE-ABS-KEY search feature; ScienceDirect results were adapted depending on the available database search options and filters. Table 2 lists all the search strategies applied to each database and the corresponding filters.

To identify more relevant research, a manual review of the reference list of relevant articles was conducted.

### 2.4. Study Selection

To organize references and detect duplicate records, all identified entries were imported into EndNote 2025.3.1 (Windows) reference management software. Before the screening, duplicate records were eliminated using the software and subsequently verified manually by two independent reviewers (A.S. and F.C.) in July 2026.

The titles and abstracts of the retrieved records were independently screened by the two reviewers relying on the established eligibility criteria. Following this, full-text publications of possibly qualified research were collected and evaluated for inclusion. A third reviewer (V.Z) monitored any conflicts between the reviewers through argumentation and agreement.

### 2.5. Data Collection and Data Items

Using a predetermined data extraction form, the two reviewers independently extracted the data. Bibliographic information (author and year of publication), study design, samples, orthodontic management, treatment techniques, and published findings were within the information retrieved. Orthodontic assessment and treatment planning, fixed appliance and restorative phased methods, expansion and introspective orthodontics and complex or surgical orthodontic management were the classifications employed to group the included research based on their chosen field of orthodontic emphasis.

### 2.6. Risk of Bias Assessment and Data Synthesis

Conventional risk of bias strategies designed for randomized or comparative studies were determined as unsuitable given that case reports and descriptive studies prevailed among the included literature. Throughout the qualitative synthesis, the methodological features and limitations of the included studies were taken into account. The diversity of study types, patient characteristics, the different therapies and documented results culminated in a qualitative synthesis of the findings. Because of the relatively small number of studies and the inconsistent nature of the existing evidence, a meta-analysis was not carried out [17].

## 3. Results

### 3.1. Study Selection

The literature search at the beginning identified 640 records from PubMed (*n* = 60), Scopus (*n* = 118) and ScienceDirect (*n* = 462). As part of the structured literature research, a 10-year publication filter was selected (2016–2026). Nevertheless the possible effect of this constraint on the depth of the available data was taken into account. After that, the database identified 257 records from PubMed (*n* = 29), Scopus (*n* = 46) and ScienceDirect (*n* = 182), in which an additional filter of Medicine and Dentistry was applied for further precision. There were 230 records left for title and abstract screening. Eleven papers were included in the qualitative synthesis after screening and full-text analysis in conformity with the predefined eligibility criteria. Figure 1 indicates the study selection process.

### 3.2. Characteristics of Included Studies

In total 11 studies were included. They were published between 2016 and 2026, and they contained mostly case reports (*n* = 10) and one retrospective descriptive clinical and cephalometric study (*n* = 1). The case reports contained individuals diagnosed with amelogenesis imperfecta; on the other hand, the retrospective descriptive clinical and cephalometric study reviewed a larger group of patients with amelogenesis imperfecta.

The reported orthodontic interventions differed based on clinical requirements and the severity of malocclusion. Orthodontic evaluation and planning, fixed orthodontic appliances, and interdisciplinary restorative-orthodontic management in coordination with surgical treatment were within the therapy techniques. Table 3, Table 4, Table 5 and Table 6 provide an overview of the features and conclusions of the selected studies.

**Figure 1:**
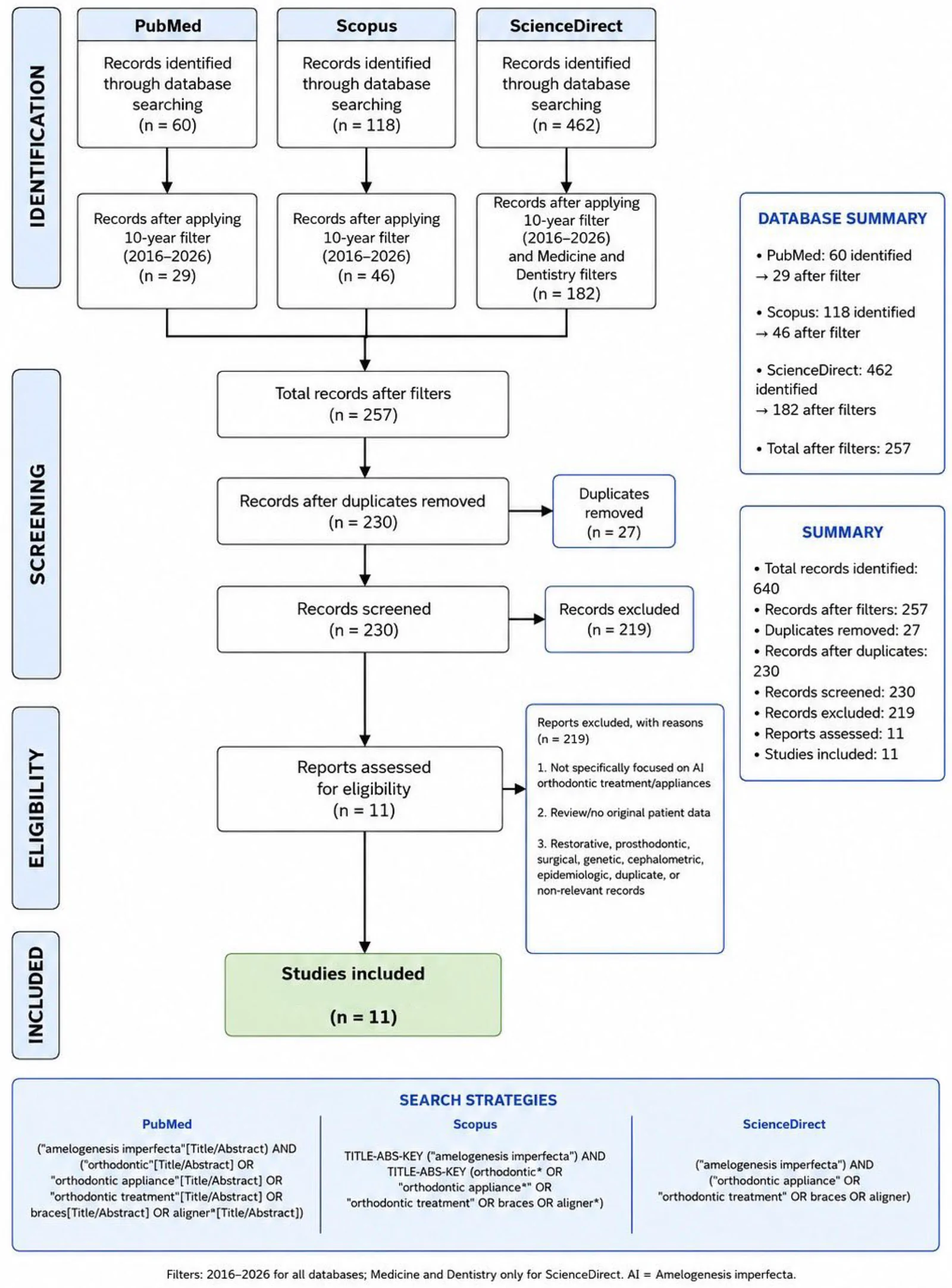
Flowchart illustrating the study selection process including article identification, screening, eligibility and inclusion.

**Table 3:** Orthodontic assessment study and treatment planning (*n* = 3).

Author (Year)	Study Design	Sample	Orthodontic Management (Treatment and Appliances)	Key Findings
Möhlhenrich et al. (2024) [3]	Retrospective descriptive clinical and cephalometric study.	Total of 19 amelogenesis imperfecta patients (mean age: 12.27 years).	Not an interventional study, no standardized orthodontic treatment protocol reported. Patients received diagnostic orthodontic assessments (study models, cephalograms, intraoral/extraoral photos) and were evaluated for treatment needs using KIG classification.	High prevalence of open bites and eruption disturbances, tendency toward dolichofacial pattern, no consistent association between AI subtype and craniofacial characteristics.
Abd Alraheam et al. (2020) [18]	Case report and short clinical review.	One patient (18-year-old male with hypoplastic amelogenesis imperfecta).	Fixed orthodontic treatment using brackets in the definitive phase after composite resin build-ups. Orthodontics used to correct malocclusion, close diastemas, extrude canines, and create inter-radicular space for restorative access. Orthodontic treatment preceded by additive composite rehabilitation of all teeth to restore vertical dimension. Retention with orthodontic retainer post-treatment.	Conservative staged multidisciplinary approach (operative dentistry, composite then orthodontic and in the end prosthodontics) is effective in young AI patients. Bonding to a well-mineralized hypoplastic enamel is relatively predictable. Adding composite restoration helps control sensitivity, restore function, and stabilize occlusion before orthodontic treatment. Full crowns may be postponed for young adults because of pulpal risk and cost.
Araujo (2025) [19]	Case report.	One patient (young male patient with amelogenesis imperfecta specifically enamel hypoplasia) in mixed dentition.	A comprehensive orthodontic treatment using modified bonding protocols due to defective enamel adhesion, alignment, leveling, diastema closure and anterior open bite correction was achieved by the use of fixed orthodontic mechanics, then periodontal crown lengthening and composite veneer rehabilitation was performed	The modified bonding technique helped with successful bracket adhesion on the amelogenesis-imperfecta-affected enamel. Orthodontic correction enhanced a stable foundation further used for restorative rehabilitation. Interdisciplinary treatment improved occlusion, esthetics, function and patient confidence.

**Table 4:** Fixed appliance and restorative staged cases (*n* = 2).

Author (Year)	Study Design	Sample	Orthodontic Management (Treatment and Appliances)	Key Findings
Jha et al. (2024) [4]	Case report.	One patient (18-year-old female with hypoplastic amelogenesis imperfecta and crowding).	Fixed orthodontic therapy using 0.022″ edgewise brackets and sequential NiTi and stainless steel archwires (0.012″ and then 0.019 × 0.025″). Bands placed on molars and buccal tubes used. Multiple rebonding episodes due to weak enamel bond strength. Standard acid etch (37% phosphoric acid, 30 s) with composite-based adhesive. Retention of fixed appliances despite enamel fragility, careful debonding using mechanical technique. Followed by prosthodontic rehabilitation (veneers, crowns).	AI causes serious orthodontic challenges due to weak enamel and frequent bracket failure. Fixed appliances can still be successful by modifying bonding protocols. Multidisciplinary approach restores function and esthetic. The patient had improved occlusion and quality of life after the treatment completion.
Leung et al. (2018) [20]	Case report.	One patient (27-year-old male with hypoplastic amelogenesis imperfecta and mild malocclusion).	Pre-prosthetic orthodontic phase to facilitate restorative work. Used rapid palatal expander for maxillary expansion and Crozat appliance for arch development. Additional fixed orthodontics used for proclination of incisors, derotation of canines, and correction of posterior cross-bites and reverse overjet. No change in OVD planned.	A multidisciplinary approach (orthodontics and prosthodontics) enabled conservative tooth preparation and improved occlusion. Achieved functional and esthetic rehabilitation with stable results. Orthodontics was essential to create interproximal space and facilitate prosthetic crowns and veneers.

**Table 5:** Expansion and interceptive orthodontics (*n* = 2).

Author (Year)	Study Design	Sample	Orthodontic Management (Treatment and Appliances)	Key Findings
Véliz et al. (2024) [9]	Case report.	One male patient (JEB due to LAMB3 mutation, syndromic generalized hypoplastic AI, followed from early childhood for 18 years).	Mini-implant-assisted rapid palatal expansion (MARPE) with 4 palatal mini-implants and 2 sequential expansion devices. Followed by fixed metallic multibracket orthodontics (19 months). Archwire sequence NiTi then stainless steel and then braided finishing wires. Additional mechanics for cross-bite correction, crowding management, and space closure extraction of #35.	MARPE successfully corrected maxillary compression and cross-bite without mucosal complications. Orthodontic phase enabled functional occlusion and space redistribution for prosthetics. Final digital rehabilitation with CAD/CAM feldspathic crowns improved esthetics, oral function, and quality of life. Multidisciplinary long-term approach (preventive, orthodontic and prosthetic) was effective in severe syndromic AI associated with JEB.
Ierardo et al. (2017) [21]	Case report	One pediatric patient (6-year-old female) with hypoplastic amelogenesis imperfecta	Modified rapid maxillary expander (RME) designed to overcome poor enamel structure, interceptive orthodontic treatment for cross-bite correction (transverse maxillary deficiency).	Successful maxillary expansion in 15 days, the results were stable on the 1-year follow-up, the modified appliance allowed effective treatment despite enamel defects

**Table 6:** Open bite and surgical/complex orthodontic cases (*n* = 4).

Author (Year)	Study Design	Sample	Orthodontic Management (Treatment and Appliances)	Key Findings
Labidi et al. (2024) [5]	Case report.	One patient (14-year-old female) with hypoplastic AI and severe anterior open bite and skeletal Class II.	Pre-orthodontic temporary crowns for bonding support, fixed orthodontic appliances used for maxillary and mandibular arch expansion (3 mm maxilla, 2 mm mandible) coordinated with orthognathic surgery (Le Fort impaction and genioplasty), braced were not bonded to natural enamel due to fragility.	Multidisciplinary approach (periodontics–orthodontics–orthognathic surgery–prosthodontics) successfully corrected severe open bite, skeletal discrepancy and esthetics, emphasized on the need for staged approach and long-term treatment stability.
Mori et al. (2019) [6]	Case report.	One patient (37-year-old male) with skeletal open bite and severe amelogenesis imperfecta).	Fixed edgewise orthodontic appliances with corticotomy and compression osteogenesis using skeletal anchorage (anchor plates at zygomatic buttress and hard palate) to intrude posterior maxilla, followed by full-mouth prosthodontic rehabilitation with provisional crowns.	Combined orthodontic, compression osteogenesis, and prosthodontic treatment effectively corrected severe skeletal open bite in amelogenesis imperfecta with stable results maintained over 8 years.
Millet et al. (2024) [7]	Case report.	One patient (10 year old female with 5-year follow-up) with severe malocclusion and amelogenesis imperfecta. She had no relevant medical history and no similar dental problems in her parents or siblings.	Preformed stainless-steel and polycarbonate crowns were placed before orthodontics. At 12.5 years, fixed bimaxillary multibracket appliances were used for presurgical orthodontic decompensation. Brackets were bonded to provisional crowns rather than defective hypoplastic enamel. Orthognathic surgery included Le Fort I osteotomy with palatal expansion and bilateral sagittal split osteotomy. A transpalatal arch and tooth positioners were used for stability/retention.	Orthodontic bonding was easier and less unpredictable when brackets were bonded to provisional crowns. Interdisciplinary treatment improved occlusion, function, esthetics, and quality of life. Final CAD/CAM zirconia crown rehabilitation remained successful with no technical or biological complications after 60 months.
Masoud et al. (2021) [8]	Case report.	One patient (16-year-old female) with hypocalcified amelogenesis imperfecta and severe anterior open bite.	Non-surgical orthodontic treatment using multiloop edgewise archwire (MEAW); 0.018 × 0.025-inch stainless steel archwires; vertical elastics, Class II elastics, reverse/compensating curves of Spee; molar intrusion and incisor extrusion mechanics; Hawley retainers for retention.	Successful correction of a 9 mm anterior open bite, improved skeletal relationships (reduced ANB and mandibular plane angle), counterclockwise mandibular rotation; stable results at 78-month follow-up, high compliance essential for success.

### 3.3. Results of Studies

The orthodontic problems mentioned in the included studies could be divided into dentoalveolar and skeletal problems. Dentoalveolar problems consisted of tooth crowding, spacing and malalignment, which were mainly treated with conventional fixed orthodontic appliances and other tooth-supported mechanics [4,7,22]. In contrast, skeletal problems were maxillary transverse deficiency, posterior cross-bite and anterior open bite. Treatment approaches could be expansion appliances or skeletal anchorage. In particular, MARPE and modified expansion appliances offer opportunities to reduce or eliminate reliance on compromised enamel for appliance support [9,10]. The clinical relevance of differentiating between dentoalveolar and skeletal problems is that skeletal corrections may permit the use of appliances with skeletal or modified support. Conventional tooth-leveling systems such as fixed braces necessarily rely on dental support and may be associated with greater bonding difficulties in teeth with defective enamel.

Early orthodontic assessment and appropriate treatment planning have a crucial role in the management of patients with amelogenesis imperfecta. Patients with AmI do not show a single orthodontic pattern; they present with a variety of malocclusions and orthodontic treatment needs such as open bites, eruption disturbances, and altered growth structures [3]. Therefore, orthodontic treatment planning should be individualized to each patient’s needs, based on a well-thought-out assessment [19].

The multidisciplinary approach is emphasized, especially in cases of enamel fragility, sensitivity, and/or reduced vertical dimension, which complicate orthodontic treatment [19]. The use of restorative build-ups or protective restorations is important before orthodontic treatment to improve function, reduce sensitivity, and generally create a more stable environment for the teeth [18], which leads to better manageable orthodontic treatment, with reduced risks associated with weak enamel. 

Modification of conventional orthodontic bonding was also suggested for more predictable results. The need for adapted protocols in patients with compromised enamel is further supported, especially when correcting open bites, spacings, and diastemas before final restorative treatment [7,19].

Furthermore, the use of fixed orthodontic appliances in patients with AI has shown to be successful; however, treatment becomes more challenging due to the quality and bond strength of the enamel [4,20]. Bracket failure and rebonding were the main clinical problems reported, especially when brackets were bonded directly to hypoplastic or weak enamel. This suggested that fixed appliances require careful bonding techniques, monitoring, and debonding procedures.

Pre-prosthetic orthodontics that are used to improve arch form, correct overbite and create adequate restorative space [4,20] can facilitate final restorative work in a later stage to be more conservative and functional. Therefore, fixed orthodontic appliances are helpful not only for improving occlusion issues, but also for making a stable environment for long-term restorations [4,20].

With an emphasis on initial management and early diagnosis of transverse discrepancies and emerging malocclusions, interceptive and expansion-related orthodontic methods have been primarily utilized in a growing number of patients with amelogenesis imperfecta [9,21]. These strategies have proved to be efficient and practical especially under conditions with structurally weak enamel. The two main approaches used for the management of maxillary constriction and posterior cross-bite were rapid maxillary expansion (RME) and mini-implant-assisted rapid palatal expansion (MARPE) [9,21]. Modified appliance designs and alternative attachment systems were implemented to eliminate the dependence on enamel bonding and limit the possibility of structural breakdown while trying to alleviate the restrictions correlated to poor enamel [9,21].

Other beneficial findings, such as the stability of treatment results during the follow-up and the satisfactory correction of transverse defects within a limited therapy duration, were frequently reported [9,21]. MARPE alongside fixed orthodontic appliances provided comprehensive care involving alignment, space redistribution and cross-bite correction without significant complications in more severe presenting conditions, like syndromic amelogenesis imperfecta related to junctional epidermolysis bullosa [9,21]. In addition, initial preventive treatment reduced the extent of future orthodontic concerns and enhanced arch growth as well as established an increasingly favorable occlusal and spatial environment, rendering restorative and prosthodontic procedures smoother in the future [9,21].

Anterior open bite and complex skeletal discrepancies are frequent in the treatment of severe malocclusions in patients with AI requiring thorough and multidisciplinary treatment methods [5,6,7,8]. The combination of skeletal instability and damaged enamel structure renders these cases classified as one of the most challenging clinical scenarios. Specifically, AI patients were often seen and associated with vertical growth patterns and skeletal Class II relationships [5,6,7,8]. According to the degree of severity, various therapeutic strategies were used. To offer the most effective treatment in cases with significant skeletal discrepancies, combined orthodontic and orthognathic surgical approaches were used [5,6,7,8]. Le Fort I osteotomy, maxillary impaction, palatal expansion, genioplasty and bilateral sagittal split osteotomy were the most common surgical methods [5,6,7,8]. With the goal to enhance bond stability, fixed appliances were frequently bonded to temporary crowns instead of natural enamel throughout presurgical orthodontic preparation [5,6,7,8].

Multiloop edgewise archwire (MEAW) mechanics paired with vertical Class II elastics enabled the effective management of severe anterior open bite through controlled molar intrusion and incisor extrusion in a handful of reported non-surgical cases [5,6,7,8]. While this approach demonstrated excellent long-term stability, it required a high level of compliance among patients [5,6,7,8].

Pre-orthodontic restorative procedures, consisting of stainless-steel crowns or polycarbonate crowns, were frequently utilized to protect the underlying tooth structure and provide a strong bonding surface due to the compromised enamel surface [5,6,7,8]. Stable functional and esthetic results were observed in a long-term follow-up, specifically when standard therapy was offered in a multimodal environment [5,6,7,8]. Comprehensive prosthodontic rehabilitation, usually containing CAD/CAM zirconia or feldspathic crowns, was crucial for preserving optimum treatment outcomes and elevating patients’ quality of life [5,6,7,8].

In general, such outcomes suggest that when therapy is individualized and supported by interdisciplinary cooperation both surgical and non-surgical techniques are successful in treating complex amelogenesis imperfecta malocclusions [5,6,7,8].

## 4. Discussion

A comprehensive overview of orthodontic management in amelogenesis imperfecta (AmI) can be obtained from the 11 included studies, which comprise case reports, case series, and observational descriptive studies [3,4,5,6,7,8,9,18,19,20,21].

The predominance of these study designs reflects the limited availability of high-level evidence regarding orthodontic treatment outcomes in this patient population. Despite variations in study designs, sample sizes, treatment protocols, and follow-up duration, the available literature consistently indicates that AmI is a complex clinical condition requiring an individualized and interdisciplinary orthodontic approach [10,23].

Evaluation of the available data indicates differences in the strength and nature of findings. Case reports provide detailed descriptions of specific orthodontic techniques and clinical modifications required for successful outcomes, whereas observational studies offer broader insight into the prevalence of malocclusions, treatment challenges, and clinical characteristics associated with AmI [3,23]. This differentiation between the available studies emphasizes the additional role of different study designs, where smaller studies contribute to individualized clinical understanding and larger studies enhance generalizability and pattern recognition [10].

The reviewed literature demonstrates that AmI is frequently associated with complex orthodontic problems, including anterior open bites, posterior cross-bites, transverse discrepancies, delayed or abnormal eruption patterns, and reduced vertical dimension [5,6,8,21]. These malocclusions are often exacerbated by enamel hypomineralization, increased sensitivity, and compromised tooth structure [5,6,8,21]. Compared with conventional orthodontic patients, the coexistence of enamel defects and malocclusion presents unique diagnostic and therapeutic challenges that require modifications in treatment planning and appliance selection [5,6,8,21].

One of the most important orthodontic considerations identified is reduced bonding reliability [4,19]. The altered mineral composition and structural integrity of AmI-affected enamel reduce adhesive capacity, increasing the risk of bracket failure during treatment [4,19,23]. Several studies emphasize the importance of modified bonding protocols, including enhanced enamel preparation, the use of alternative adhesive systems, and close monitoring throughout treatment [7,19,24]. These findings suggest that although orthodontic tooth movement is feasible, conventional bonding strategies often require adaptation to improve treatment predictability [7,23,24].

When comparing AmI with other developmental dental anomalies, both similarities and differences become evident [11,12]. Dentinogenesis imperfecta (DI), for example, is also a hereditary condition affecting dental tissues; however, it primarily involves dentin rather than enamel [11,25]. This distinction has important biomechanical implications, as DI is associated with increased susceptibility to fractures under orthodontic forces, whereas AmI presents challenges mainly related to bonding and enamel integrity [11,25]. Therefore, while both conditions require interdisciplinary management and careful force control, the underlying structures differ significantly [12,14,15,26,27]. Anomalies, such as microdontia and macrodontia, also influence orthodontic treatment but through other mechanisms [26,27]. According to Endo et al. microdontia typically leads to spacing and esthetic concerns requiring combined orthodontic and restorative approaches, while macrodontia often results in crowding and may necessitate space-gaining strategies or extractions [28]. Unlike AI, these conditions generally do not involve compromised enamel bonding, highlighting the importance of tissue quality in determining orthodontic complexity [26,28].

Based on Jafarzadeh et al. additional anatomical variations, including taurodontism, which is frequently associated with AmI, may further complicate orthodontic management [29,30]. Altered root morphology and enlarged pulp chambers can affect biomechanics, extraction planning, and the risk of endodontic complications [29,30]. Referring to Shifman & Chanannel et al. consequently, thorough radiographic assessment is essential for accurate diagnosis and treatment planning in these patients [29,30].

The literature also shows the importance of a multidisciplinary approach [18,20]. Orthodontic treatment in AmI patients is closely linked to restorative and prosthodontic needs, as enamel defects often result in esthetic concerns, tooth sensitivity, and reduced crown integrity [7,24]. Coordinated treatment planning involving orthodontists and restorative specialists improves both functional and esthetic outcomes and allows for more efficient sequencing of care [5,9]. Furthermore, pediatric dentists have a crucial role in early diagnosis, preventive measures, enamel breakdown monitoring and proper oral care maintenance specifically during orthodontic treatment [31,32].

Oral hygiene should also receive special attention. The presence of orthodontic appliances and the uneven and structurally damaged tooth surfaces linked to AmI may increase plaque retention and complicate efficient plaque control [32]. Inadequate oral hygiene might exacerbate periodontal and enamel weakness. Therefore, preventive care, routine professional monitoring, and customized oral hygiene teaching should be regarded as essential elements of orthodontic treatment for individuals with Aml [3,9].

The timing of treatment is another important factor influencing outcomes [9,21]. Early diagnosis and interceptive management may reduce the severity of developing malocclusions, particularly in cases involving eruption disturbances and transverse discrepancies [9,21]. However, due to variability in disease severity and the limited number of long-term studies, determining the optimal timing of intervention remains challenging [3,9].

In patients with AmI, dentoalveolar issues such as crowding, spacing, and tooth malalignment are typically treated with traditional fixed orthodontic appliances, but bonding and appliance retention are still difficult due to compromised enamel, according to recent research [4,7,22]. On the other hand, skeletal issues such as posterior cross-bites, maxillary transverse deficits, and severe vertical discrepancies can be treated with skeletal anchors, expansion appliances, or a combination of orthodontic and surgical techniques [5,6,7,11]. Because they can lessen reliance on weakened enamel for appliance support, MARPE and modified expansion designs in particular constitute a significant recent development [9,11]. As a result, current treatment trends stress customized appliance selection based on whether the primary issue is skeletal or dentoalveolar, giving skeletal or modified support more weight when enamel quality limits traditional tooth-borne mechanics.

Despite generally favorable clinical outcomes, several limitations must be considered [3,4,8]. Based on Masoud et al. the predominance of case reports and small case series limits the generalizability of findings [3,4,8]. Additionally, there is considerable heterogeneity in treatment protocols, bonding techniques, follow-up periods, and outcome measures across studies [3,4,8]. These variations make direct comparison difficult and hinder the development of standardized clinical guidelines [3,4,8].

Furthermore, important variables are not consistently evaluated across studies [3,9,18]. Factors such as AmI subtype, genetic background, severity of enamel defects, patient age, oral hygiene status, and prior restorative treatment may significantly influence treatment outcomes but remain underreported in the literature [3,9,18]. The predominance of case reports and small observational studies makes it difficult to reliably compare how well different treatment approaches work and limits the ability to figure out whether any particular intervention is superior. Additionally, the limited availability of long-term follow-up data makes the stability and durability of reported treatment outcomes uncertain. Publication bias should be considered, as successful or unique clinical cases are more likely to be reported.

Overall, the available evidence supports a multifactorial understanding of orthodontic management in AmI [3,8]. Treatment outcomes are influenced by enamel characteristics, malocclusion severity, eruption disturbances, and patient-specific factors [3,4,18]. Successful management requires careful diagnosis, modified bonding techniques, individualized biomechanics, and continuous interdisciplinary collaboration [4,8,9].

Future research should focus on improving evidence quality and treatment predictability. Well-designed prospective clinical studies evaluating standardized treatment protocols, long-term stability, and the effectiveness of modern adhesive systems in AmI patients are needed. Advances in biomaterials, digital orthodontics, and interdisciplinary care models may further improve treatment predictability and patient outcomes.

In conclusion, the current evidence indicates that orthodontic treatment in patients with Aml presents unique challenges compared with other dental anomalies due to the combination of enamel structural defects and complex malocclusions. Although available studies are limited in level of evidence, they consistently support an individualized, interdisciplinary approach to achieve predictable functional and esthetic outcomes. Early diagnosis, modified orthodontic techniques, attention to oral hygiene and periodontal health, coordinated pediatric dental, orthodontic, restorative, prosthodontic and periodontal care, and coordinated restorative planning remain essential components of successful AmI management.

## 5. Conclusions

According to the available research, patients with amelogenesis imperfecta experience substantial clinical difficulties during orthodontic treatment because of enamel structural defects, weakened bonding and the high incidence of complex malocclusions. Modified orthodontic methods and careful biomechanical planning customized for each patient are usually essential for efficient therapy.

Proper assessment, individualized treatment planning and a comprehensive approach that contains orthodontic, restorative and prosthodontic cooperation are strongly connected to favorable results. Additional procedures such orthognathic surgery or skeletal anchoring might be essential in more complicated cases in order to provide the most optimal and functional cosmetic outcomes.

However, the available evidence remains minimal and primarily gathered from case reports and small observational studies. Therefore, the existing literature supports the clinical feasibility of these approaches rather than their effectiveness or superiority. More carefully organized longitudinal and clinical research is required to strengthen the collection of data and promote the formulation of more standardized treatment plans.

Suggestions for conclusion as bullet points include:

- Patients with amelogenesis imperfecta commonly present with complex orthodontic problems, including bonding difficulties, eruption disturbances, transverse discrepancies and anterior open bites.
- Modified bonding techniques, alternative appliance designs and individualized planning were described recurrently across studies.
- Restorative build-ups, skeletal anchorage, expansion procedures and combined orthodontic–orthognathic treatment were reported in selected cases according to individual clinical requirements.
- Orthodontic treatment was commonly planned in conjunction with restorative and prosthodontic care.
- The available evidence is derived predominantly from case reports, case series and small observational studies, and therefore demonstrates clinical feasibility rather than comparative effectiveness or superiority of any particular treatment approach.

## Figures and Tables

**Table 2:** Database-specific search strategies used for the literature search and the applied filters.

Database	Search Strategy	Filters Applied
PubMed	(“amelogenesis imperfecta” [Title/Abstract]) AND (orthodontic* [Title/Abstract] OR “orthodontic appliance*” [Title/Abstract] OR “orthodontic treatment” [Title/Abstract] OR braces [Title/Abstract] OR aligner* [Title/Abstract])	Publication years: 2016–2026
Scopus	TITLE-ABS-KEY (“amelogenesis imperfecta”) AND TITLE-ABS-KEY (orthodontic* OR “orthodontic appliance*” OR “orthodontic treatment” OR braces OR aligner*)	Publication years: 2016–2026
ScienceDirect	(“amelogenesis imperfecta”) AND (“orthodontic appliance” OR “orthodontic treatment” OR braces OR aligner)	Publication years: 2016–2026 Subject area: Medicine and Dentistry

## Data Availability

The original contributions presented in this study are included in the article. Further inquiries can be directed to the corresponding authors.
